# Real-world Practice Patterns and Barriers to SGLT2 Inhibitor Use: A Nationwide Survey of Japanese General Practitioners

**DOI:** 10.31662/jmaj.2026-0197

**Published:** 2026-08-21

**Authors:** Satoru Tatematsu, Kazuo Kobayashi, Tsuguru Hatta, Taisuke Isozaki, Yosuke Nakayama, Junko Imura, Toshimasa Takahashi, Munehiro Kitada, Yasunori Utsunomiya

**Affiliations:** 1 Committee of Kidney and Electrolyte Disease, Japan Physicians Association, Tokyo, Japan; 2 Division of Nephrology, Department of Internal Medicine, Edogawa Medicare Hospital, Tokyo, Japan; 3 Department of Medical Science and Cardiorenal Medicine, Yokohama City University Graduate School of Medicine, Kanagawa, Japan; 4 Hatta Internal Medicine Hospital, Kyoto, Japan; 5 Isozaki Family Clinic, Shizuoka, Japan; 6 Iyasaka Kidney Clinic, Hiroshima, Japan; 7 Imura Internal Medicine and Dialysis Clinic, Kanazawa, Japan; 8 Bosei Shinjuku Southgate Clinic, Tokyo, Japan; 9 Hamada Medical Clinic, Wakayama, Japan; 10 Hoya Hospital, Tokyo, Japan

**Keywords:** chronic kidney disease, general practitioner, questionnaire survey, sodium–glucose cotransporter inhibitors

## Abstract

**Introduction:**

Sodium–glucose cotransporter 2 (SGLT2) inhibitors were approved for the treatment of chronic kidney disease (CKD) in Japan in 2021. However, their use remains limited, and broader implementation is necessary to improve CKD outcomes.

**Methods:**

A questionnaire survey on the diagnosis and treatment of CKD was conducted among members of the Japan Physicians Association and local internal medicine associations. Based on responses regarding the use of SGLT2 inhibitors in patients with CKD, respondents excluding nephrologists were classified into two groups: the Active User group (n = 508) and the Passive User group (n = 316). The responses were compared between groups, and independent factors associated with active use were analyzed using logistic regression analysis.

**Results:**

Compared with the Passive User group, respondents in the Active User group were younger (p = 0.047). Specialization in cardiology, allergy, use of serum cystatin C to evaluate kidney function, utilization of urine testing, use of estimated glomerular filtration rate and kidney imaging to define CKD, frequent assessment of quantitative albuminuria, evaluation of hemoglobin and transferrin saturation levels to assess renal anemia, prescription of hypoxia-inducible factor prolyl hydroxylase inhibitors, implementation of dietary therapy for patients with non-dialysis CKD, referral to nephrologists for both proteinuria and hematuria, and consultation for proteinuria >1.0 g/gCr or ≥2+ by qualitative testing were all positively associated with the Active User group, with odds ratios of 1.84, 6.13, 2.05, 34.8, 1.96, 4.52, 1.72, 2.10, 2.56, 2.03, and 2.62, respectively.

**Conclusions:**

Among non-nephrologists, difference in the active prescription of SGLT2 inhibitors for patients with CKD was associated with a comprehensive and guideline-based approach to CKD management.

## Introduction

Chronic kidney disease (CKD) is recognized as a persistent global public health challenge, affecting approximately 10% of adults and conferring significant risks for end-stage kidney disease (ESKD), cardiovascular events, and premature mortality ^(1)^. In Japan, the prevalence of CKD is rising rapidly, largely due to an aging population and the increasing burden of diabetic kidney disease, which remains the leading cause of dialysis initiation ^(2), (3)^. Conventional strategies, including optimal blood pressure and glycemic control and the use of renin–angiotensin system (RAS) inhibitors for CKD with proteinuria, have modestly reduced the incidence of ESKD; however, many patients still progress to kidney failure despite maximal therapy ^(4), (5)^.

The introduction of sodium–glucose cotransporter 2 (SGLT2) inhibitors has substantially transformed CKD management. Originally developed for type 2 diabetes, SGLT2 inhibitors have demonstrated robust renoprotective and cardiovascular benefits in several large-scale randomized controlled trials, including CREDENCE ^(6)^, DAPA-CKD ^(7)^, and EMPA-KIDNEY ^(8)^. These agents have consistently shown reductions in CKD progression, dialysis initiation, and cardiovascular events in both diabetic and non-diabetic populations. The Kidney Disease: Improving Global Outcomes (KDIGO) and the Japanese Society of Nephrology guidelines now recommend SGLT2 inhibitors as core therapy for patients with CKD and albuminuria, irrespective of diabetes status ^(9), (10)^.

Nevertheless, real-world use of SGLT2 inhibitors among eligible patients with CKD remains low in Japan and globally, despite compelling clinical evidence ^(11)^. Japan Physicians Association performed nationwide questionnaire survey to clarify the awareness of general practitioners (GPs) for the CKD management. Our survey raised concerns about the low use of guidelines and implementation of quantitative proteinuria measurements among non-nephrologists ^(12)^ and discrepancies in CKD management between nephrologists and non-nephrologists, with insufficient regional CKD management–related collaboration ^(13)^. This study is a post-hoc analysis using the nationwide questionnaire survey conducted to identify real-world determinants of SGLT2 inhibitor prescription in CKD management. Building on global evidence gaps and major trial outcomes, this research aims to elucidate modifiable provider-, practice-, and organization-level factors that could promote the appropriate use of SGLT2 inhibitors. The findings are expected to support tailored interventions that enhance guideline adherence, foster multidisciplinary collaboration, and improve uptake of nephroprotective therapies for patients with CKD in Japan and comparable healthcare systems.

## Materials and Methods

### Participants, survey, and procedures

This questionnaire survey was conducted by the Japan Physicians Association, a nationwide organization comprising 13,014 GPs working in hospitals and clinics across Japan as of May 2023. The questionnaire was mailed to all members of the Japan Physicians Association in April 2024. A total of 1,003 members (approximately 7.7%) voluntarily responded between April and October 2024 and participated in this survey. The 2024 survey included 20 questions related to the diagnosis, treatment, and management of patients with CKD, and 10 questions concerning CKD collaborative care between GPs and nephrologists. Because the level of activeness in prescribing SGLT2 inhibitors for patients with CKD was found to be lower among non-nephrologists than that among nephrologists in our previous study ^(12)^, this post-hoc analysis focused exclusively on respondents who were not nephrologists. Supplementary Table 1 presents the distribution of responses to the question on SGLT2 inhibitor use in patients with CKD (Question 15 [Q15]). This post-hoc survey focused on analyzing whether respondents use SGLT2 inhibitors as new medications for CKD or as a treatment only for patients with diabetic nephropathy. Accordingly, respondents who answered either “I prescribe them to patients with CKD regardless of the presence or absence of proteinuria/albuminuria” or “I prescribe them to patients with CKD and positive proteinuria/albuminuria” were categorized as the Active User group (n = 508), because both responses indicate that CKD itself is considered a treatment target. In contrast, respondents who answered “I prescribe them only to patients with diabetes and positive proteinuria/albuminuria,” “I do not prescribe them because patients are diagnosed as CKD,” or “I do not prescribe them” were categorized as the Passive User group (n = 316), as these responses reflect a more restrictive prescribing attitude toward CKD.

### Statistical analysis

The characteristics of respondents and their answers to the 30 survey questions were converted into discrete variables. The chi-square test was used to compare the two groups. For comparisons involving multiple options between groups, Bonferroni correction was applied to each option following the chi-square test. Responses to Questions 1, 3, 4, 5, 6, 7, 11, 12, 13, 14, 16,18, 19, 21, 22, 23, 24, 26, 27, and 29 were converted into ordinal variables. Logistic regression analysis was conducted to identify independent variables associated with the Active User group. Stepwise method was performed, using background characteristics of respondents and responses related to CKD management and collaboration as explanatory variables (Supplementary Tabe 2).

Statistical significance was defined as p < 0.05. All analyses were performed using IBM SPSS Statistics software (version 28.0; IBM Corp., Armonk, NY, USA).

This study was conducted in accordance with the principles of the Declaration of Helsinki and was approved by the Ethical Review Board of the Japan Physicians Association on December 18, 2023 (Approval No. 0.3-2312-01).

## Results

### Characteristics of the respondents

Table 1 presents the characteristics of the respondent GPs in each group. A significant difference was observed in age distribution between the two groups (p = 0.047 by the chi-square test and p = 0.002 by the Cochrane–Armitage test), with the Active User group including a higher proportion of respondents in their 50s and the Passive User group including more respondents aged 70 years and older. The proportion of cardiologists, allergists, and those with a history of nephrology training was significantly higher in the Active User group than in the Passive User group (p < 0.001 and p = 0.01, respectively). Our previous report demonstrated that nephrologists prescribed SGLT2 inhibitors for patients with CKD more frequently than non-nephrologists ^(12)^.

**Table 1.** Characteristics of the Respondent GPs According to the Utilization of SGLT2 Inhibitors.

Variable			Active user group	Passive user group	p Value
			(n = 508)	(n = 316)	
Age distribution		20s	1 (0.2)	0 (0.0)	0.047
		30s	10 (2.0)	3 (0.9)	
		40s	36 (7.1)	20 (6.3)	
		50s	127 (25.0)^＊^	58 (18.4)^＊^	
		60s	205 (40.4)	126 (39.9)	
		70 years and older	129 (25.4)^＊^	108 (34.2)^＊^	
		No answer	0 (0.0)	1 (0.3)	
Workplace	Type	Clinic	422 (83.0)	259 (81.9)	0.15
		Hospital	85 (16.8)	55 (17.4)	
		No answer	1 (0.2)	2 (0.6)	
	Region in Japan	Hokkaido/Tohoku	48 (9.5)	14 (5.1)	0.10
		Kanto	154 (30.7)	79 (25.2)	
		Chubu/Hokuriku	69 (14.1)	53 (16.9)	
		Kinki	85 (17.4)	62 (20.1)	
		Chugoku/Shikoku	65 (13.5)	44 (14.3)	
		Kyushu/Okinawa	65 (13.1)	47 (15.6)	
		No answer	9 (1.8)	9 (2.9)	
History of nephrology training		Yes	134 (26.4)^＊^	63 (19.9)^＊^	0.01
		No	374 (73.6)	250 (79.1)	
		No answer	0 (0.0)	3 (0.9)	
Specialty		Internal medicine	426 (83.9)	276 (87.3)	0.17
		Cardiology	113 (22.2)	39 (12.3)	<0.001
		Diabetology and metabolism	93 (18.3)	43 (13.6)	0.08
		Gastroenterology	96 (18.9)	63 (19.9)	0.71
		Pulmonology	41 (8.1)	18 (5.7)	0.20
		Neurology	14 (2.8)	15 (4.7)	0.13
		Hematology	12 (2.4)	7 (2.2)	0.89
		Rheumatology	12 (2.4)	10 (3.2)	0.49
		Allergy	22 (4.3)	5 (1.6)	0.03
		Psychiatry	7 (1.4)	6 (1.9)	0.56
		Other	17 (3.3)	17 (5.4)	015

Values are expressed as n (%). p values were calculated using the chi-square test. ^＊^Statistically significant differences were observed after Bonferroni correction. GP: general practitioner; SGLT2: sodium–glucose cotransporter 2.

### Comparisons of questionnaire results between the Active and Passive User groups

Table 2 and Figure 1 present the results of the questionnaire on CKD management according to the utilization of SGLT2 inhibitors. Regarding questions related to CKD evaluation, compared with the Passive User group, the Active User group showed significantly higher possession and utilization of CKD guidelines (Q1), greater use of estimated glomerular filtration rate (eGFR)_creatinine_ or eGFR_cystatinC_ levels for assessing CKD (Q2), more accurate CKD diagnosis (Q3), more frequent performance of quantitative tests for proteinuria (Q4) and albuminuria (Q5), more frequent home blood pressure evaluation (Q6), more consistent prescription of RAS inhibitors for patients with CKD and hypertension (Q8), and more frequent use of Tanaka’s formula for evaluating salt intake (Q9) (p = 0.02, p = 0.001, p < 0.001, p < 0.001, p < 0.001, p = 0.01, p < 0.001, p = 0.02, and p < 0.001, respectively).

**Table 2.** Results of Questionnaires on CKD Medical Care According to the Utilization of SGLT2 Inhibitors.

		Active user group	Passive user group	p Value†
		(n = 508)	(n = 316)	
Q1	Do you possess the CKD guidelines 2023?			
	Yes, and I use them frequently.	121 (23.8)^#^	52 (16.5)^#^	0.02
	Yes, but I have not used them.	175 (34.4)	106 (33.5)	
	No, but I would like to obtain the CKD Guidelines 2024 for GPs.	176 (34.6)	121 (38.3)	
	I do not possess them and do not wish to obtain the CKD Guidelines 2024 for GPs.	32 (6.3)^＊^	35 (11.1)^＊^	
	No answer.	4 (0.8)	2 (0.6)	
Q2*	What parameters do you use to assess kidney function?			
	Serum creatinine level.	291 (57.3)	203 (64.2)	0.048
	eGFR_creatinine_ level.	491 (96.7)	289 (91.5)	0.001
	Serum cystatin C level.	74 (14.6)	33 (10.3)	0.09
	eGFR_cystatinC_ level.	81 (15.9)	24 (7.6)	< 0.001
	Creatinine clearance (24-h urine collection).	12 (2.4)	9 (1.9)	0.66
Q3	How do you diagnose patients as having CKD?
	Only by urine test.	2 (0.4)^#^	6 (1.9)^#^	< 0.001
	Only by eGFR level.	29 (5.7)^#^	41 (13.0)^#^	
	By both urine test and eGFR level.	370 (72.8)	234 (74.1)	
	By urine test, eGFR level, and kidney imaging.	104 (20.5)^#^	33 (10.4)^#^	
	No answer	3 (0.6)	2 (0.6)	
Q4	Do you check urine tests for patients with CKD during regular visits?			
	Perform quantitative tests for proteinuria (g/gCr).	203 (40.0)^#^	170 (53.8)^#^	< 0.001
	Perform only qualitative tests for proteinuria.	205 (40.4)^#^	84 (26.6)^#^	
	Do not perform urine testing.	10 (2.0)	11 (3.5)	
	No answer	90 (17.7)	51 (16.1)	
Q5	Do you check albuminuria in patients with diabetes during regular visits?			
	Quantitative tests every 3 months.	102 (20.1)^#^	40 (12.7)^#^	< 0.001
	Quantitative tests once or twice a year.	216 (42.5)^#^	76 (24.1)^#^	
	Rarely perform quantitative tests.	75 (14.8)^#^	69 (21.8)^#^	
	Perform only qualitative tests.	32 (6.3)	30 (9.5)	
	Do not check albuminuria.	82 (16.1)^#^	100 (31.6)^#^	
	No answer	1 (0.2%)	1 (0.3)	
Q6	Do you evaluate BP in patients with CKD during clinic visits?			
	I evaluate both office BP and home BP in the early morning.	444 (87.4)^#^	259 (82.0)^#^	0.01
	I evaluate only home BP in the early morning.	27 (5.3)	14 (4.4)	
	I evaluate only office BP.	36 (7.1)^#^	38 (12.0)^#^	
	I do not evaluate either.	0 (0.0)^#^	5 (1.6)^#^	
	No answer	1 (0.2)	0 (0.0)	
Q7	Do you prescribe RAS inhibitors to patients with CKD and hypertension?			
	I prescribe them as first-line medication.	393 (77.4)^#^	222 (70.3)^#^	< 0.001
	I prescribe them to patients with proteinuria.	93 (18.3)	54 (17.1)	
	I rarely prescribe them.	20 (3.9)^#^	39 (12.3)^#^	
	No answer	2 (0.4)	1 (0.3)	
Q8*	When do you discontinue RAS inhibitors in patients with CKD?			
	When kidney function deteriorates.	178 (35.3)	125 (39.8)	0.20
	When the serum Cr level reaches 2 mg/dL.	122 (24.2)	72 (22.9)	0.68
	When the serum potassium level exceeds the normal range.	225 (44.6)	152 (48.4)	0.29
	I continue prescribing them for their renoprotective effects.	245 (48.6)	127 (40.4)	0.02
	I do not prescribe them to patients with CKD.	7 (1.4)	16 (5.1)	0.002
Q9*	How do you evaluate salt intake in patients with CKD?			
	By Tanaka’s formula using spot urine samples.	198 (40.1)	74 (24.3)	< 0.001
	By direct measurement using 24-h urine collection.	2 (0.4)	6 (2.0)	0.03
	By patient interview.	164 (33.2)	114 (37.4)	0.23
	I do not evaluate salt intake by either method.	143 (28.9)	120 (39.3)	0.002
Q10*	Which parameters do you use to evaluate renal anemia?			
	Hemoglobin level.	500 (98.4)	300 (95.2)	0.007
	Mean corpuscular volume (MCV).	301 (59.3)	144 (45.7)	< 0.001
	Serum iron (Fe) level.	362 (71.3)	194 (61.6)	0.004
	Transferrin saturation (TSAT).	162 (31.9)	47 (14.9)	< 0.001
	Serum ferritin level.	362 (71.3)	187 (59.4)	< 0.001
	Serum erythropoietin level.	232 (45.7)	148 (47.0)	0.71
Q11	Do you prescribe erythropoiesis-stimulating agents to patients with CKD?		
	Yes, I prescribe them at my own discretion.	347 (68.3)^#^	165 (52.2)^#^	< 0.001
	Yes, I prescribe them when recommended by a nephrologist.	92 (18.1)^#^	83 (26.3)^#^	
	No, I do not prescribe them.	63 (12.4)^#^	63 (19.9)^#^	
	No answer	6 (1.2)	5 (1.6)	
Q12	Do you prescribe HIF-PH inhibitors to patients with CKD?			
	Yes, I prescribe them at my own discretion.	306 (60.2)^#^	110 (34.8)^#^	< 0.001
	Yes, I prescribe them when recommended by a nephrologist.	102 (20.1)	78 (24.7)	
	No, I do not prescribe them.	87 (17.1)^#^	118 (37.3)^#^	
	No answer	13 (2.3)	10 (3.2)	
Q16	How do you manage serum potassium levels in patients with CKD?			
	I regularly check potassium levels and manage hyperkalemia continuously.	333 (65.6)^#^	150 (47.5)^#^	< 0.001
	I check potassium regularly but provide only temporary treatment.	82 (16.1)^#^	73 (23.1)^#^	
	I check potassium regularly but refer hyperkalemia management to a nephrologist.	62 (12.2)^#^	57 (18.0)^#^	
	I do not check potassium but manage hyperkalemia per nephrologist instructions.	24 (4.7)^#^	26 (8.2)^#^	
	I neither check nor treat hyperkalemia.	7 (1.4)	10 (3.2)	
Q18	Do you evaluate serum calcium and phosphorus levels in patients with CKD?			
	I regularly check both calcium and phosphorus levels.	258 (50.8)^#^	106 (33.5)^#^	< 0.001
	I regularly check only calcium levels.	119 (23.4)	82 (25.9)	
	I check them only in patients on vitamin D therapy.	65 (12.8)	39 (12.3)	
	I do not check them but rely on nephrologists.	45 (8.9)^#^	63 (19.9)^#^	
	I never check them.	16 (3.1)^#^	26 (8.2)^#^	
	No answer	5 (1.0)	0 (0.0)	
Q19	Do you provide dietary therapy to patients with non–dialysis-dependent CKD?
	I provide it at my clinic or hospital.	345 (67.9)^#^	159 (50.3)^#^	< 0.001
	I refer patients to nephrologists for dietary therapy.	107 (21.1)^#^	96 (30.4)^#^	
	I neither provide nor refer for dietary therapy.	53 (10.4)^#^	61 (19.3)^#^	
	No answer	3 (0.6)	0 (0.0)	
Q20*	What types of CKD dietary therapy do you provide?			
	Restriction of daily salt intake.	479 (96.8)	284 (94.0)	0.06
	Restriction of daily protein intake.	322 (65.1)	189 (62.6)	0.48
	Guidance on appropriate daily calorie intake.	171 (34.5)	89 (29.5)	0.14
	Restriction of daily phosphate intake.	223 (45.1)	97 (32.1)	< 0.001

Values are expressed as n (%). BP: blood pressure; CKD: chronic kidney disease; Cr: creatinine; eGFR: estimated glomerular filtration rate; GP: general practitioner; HIF-PH: hypoxia-inducible factor prolyl hydroxylase; Q: question; RAS: renin–angiotensin system; SGLT2: sodium–glucose cotransporter 2. †p values were calculated using the chi-square test. ^＊^Multiple responses were allowed where indicated. # Significant differences were observed after Bonferroni correction.

**Figure 1.**
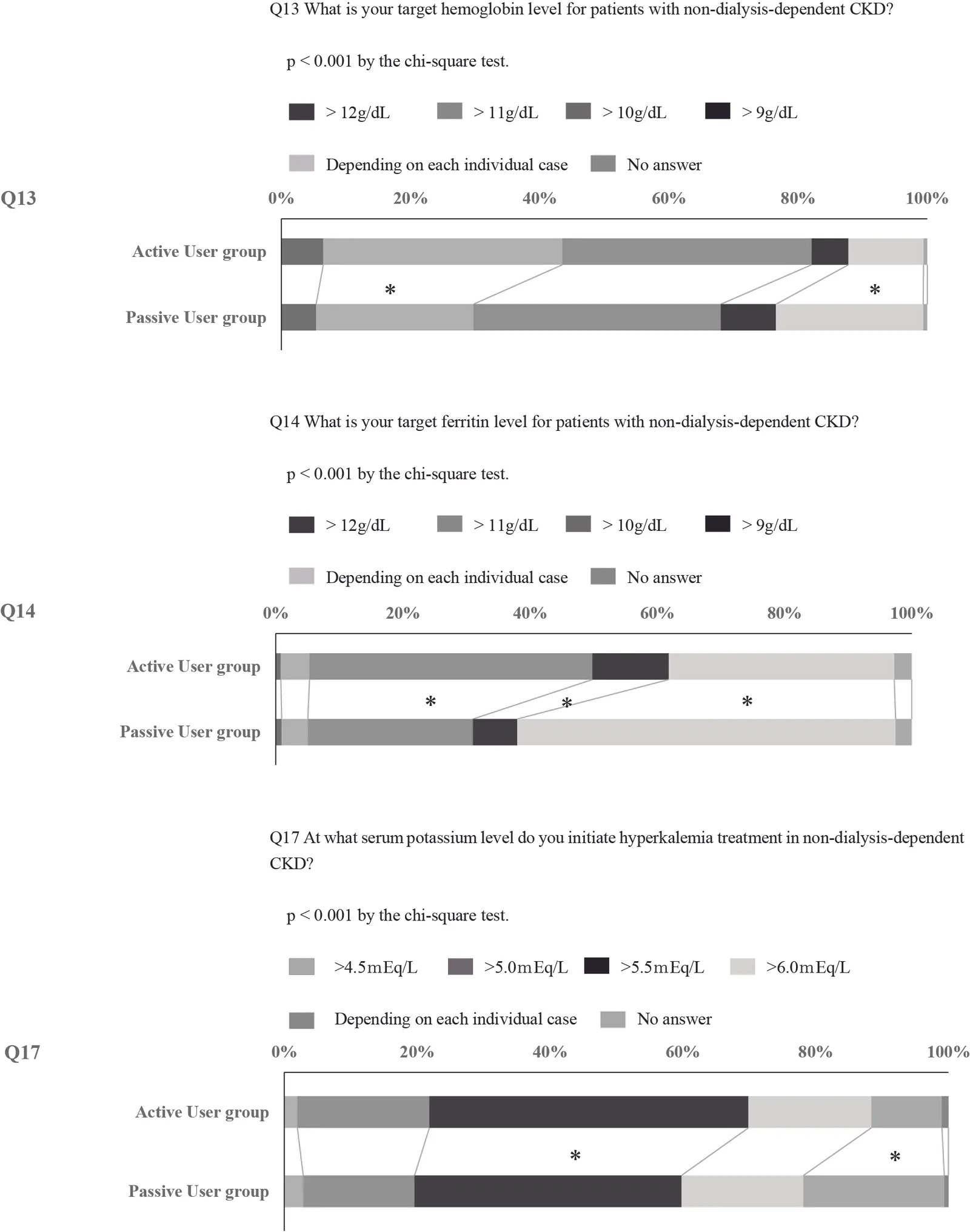
Results of questionnaires on CKD medical care according to the utilization of SGLT2 inhibitors (Q13, Q14 and Q17). ^＊^Significant differences were observed after Bonferroni correction. Abbreviations: CKD, chronic kidney disease; SGLT2, sodium–glucose cotransporter 2.

Regarding questions related to CKD treatment, compared with the Passive User group, the Active User group showed a higher prevalence of prescribing erythropoiesis-stimulating agents (Q11) and hypoxia-inducible factor prolyl hydroxylase (HIF-PH) inhibitors (Q12), a higher hemoglobin target for managing renal anemia (Q13), more frequent management of hyperkalemia (Q16) and abnormalities in serum calcium or phosphate levels (Q18), and more frequent implementation of dietary therapy for patients with CKD (Q19) (all p < 0.001).

Table 3 and Figure 2 show the results of the questionnaire regarding CKD collaborative care between GPs and nephrologists based on SGLT2 inhibitor utilization. Compared with the Passive User group, the Active User group reported more favorable relationships with nephrologists (Q23), more frequent use of referral criteria for CKD consultation (Q24), higher eGFR levels (Q26) and lower proteinuria levels (Q27) at the time of referral, and less frequent requests for the management of blood pressure, plasma glucose, and renal anemia (p = 0.003, p = 0.004, p = 0.04, p = 0.004, p = 0.001, p = 0.01, and p = 0.002, respectively).

**Table 3.** Results of Questionnaires on CKD Collaborative Care between GPs and Nephrologists According to the Utilization of SGLT2 Inhibitors.

		Active user group	Passive user group	p Value†
		(n = 508)	(n = 316)	
Q21	Is CKD collaborative care between GPs and nephrologists active in your region?			
	Yes, fully active.	128 (25.2)	71 (22.5)	0.64
	Yes, partially active.	234 (46.1)	149 (47.2)	
	No, it does not work well.	108 (21.3)	64 (20.3)	
	No, and there is no CKD collaborative care between GPs and nephrologists.	36 (7.1)	31 (9.8)	
	No answer	2 (0.4)	1 (0.3)	
Q22	Do you know any nephrologists whom you can consult regarding patients with CKD?			
	Yes, I know several nephrologists available for consultation.	385 (75.8)	241 (67.3)	0.09
	Yes, I know only one nephrologist available for consultation.	71 (14.0)	31 (9.8)	
	No	48 (9.4)	43 (13.6)	
	No answer	4 (0.8)	1 (0.3)	
Q23	How is your relationship with nephrologists available for consultation?			
	I know them well personally.	169 (33.3)^#^	71 (22.5)^#^	0.003
	I know only their names.	277 (54.5)	190 (60.1)	
	I do not know them well.	58 (11.4)^#^	54 (17.1)^#^	
	No answer	4 (0.8)	1 (0.3)	
Q24	Do you use the referral criteria for CKD consultation recommended in the CKD Guidelines (Japanese Society of Nephrology)?			
	I use it often.	86 (16.9)	38 (12.0)	0.004
	I use it sometimes.	230 (45.3)^#^	120 (38.0)^#^	
	I rarely use them.	100 (19.7)	64 (20.3)	
	I know about them but do not use them.	70 (13.8)^#^	73 (23.1)^#^	
	I do not know about them.	19 (3.7)	19 (6.0)	
	No answer	3 (0.6)	2 (0.6)	
Q25*	For which patients with CKD do you usually consult nephrologists?			
	With evident proteinuria (> 0.5 g/gCr)	294 (59.6)	187 (59.4)	0.94
	With both proteinuria and hematuria	250 (50.7)	117 (37.1)	< 0.001
	With eGFR < 45 mL/min/1.73 m^2^	313 (63.5)	218 (69.2)	0.10
	With rapid eGFR decline	420 (85.2)	264 (83.8)	0.60
	With worsening symptoms including edema	208 (42.2)	155 (49.2)	0.05
	With worsening control of blood pressure or glucose	87 (17.6)	72 (22.2)	0.07
	With need for dietary counseling	128 (26.0)	90 (28.6)	0.42
	With need for explanation of kidney replacement therapy or conservative kidney management	313 (63.5)	173 (54.9)	0.02
Q28*	What CKD-related treatments would you like nephrologists to provide?			
	Salt reduction counseling	192 (39.0)	121 (37.7)	0.92
	CKD-specific dietary counseling	326 (66.3)	221 (70.6)	0.20
	Management of blood pressure	124 (25.2)	112 (35.8)	0.001
	Management of plasma glucose	60 (12.2)	58 (18.5)	0.01
	Treatment for renal anemia	181 (36.8)	149 (47.6)	0.002
	Treatment of electrolyte abnormalities or metabolic acidosis	289 (58.7)	188 (60.1)	0.71
	Explanation of kidney replacement therapy or conservative kidney management	412 (83.7)	265 (84.7)	0.73
Q29	Are you satisfied with nephrologists’ CKD consultation?
	Completely satisfied	129 (26.1)	58 (18.4)	0.11
	Satisfied sometimes	214 (43.2)	150 (47.6)	
	Neither satisfied nor dissatisfied	114 (23.0)	84 (26.7)	
	Occasionally dissatisfied	28 (5.7)	18 (5.7)	
	Not satisfied at all	6 (1.2)	1 (0.3)	
	No answer	4 (0.8)	4 (1.3)	
Q30*	Which aspects of the consultation were unsatisfactory?			
	Insufficient patient counseling or explanation	60 (12.2)	34 (10.9)	0.57
	Insufficient feedback to GPs	83 (16.8)	51 (16.3)	0.84
	Inadequate treatment (e.g., no change in medication)	99 (20.1)	59 (18.8)	0.67
	Insufficient counseling by nutritionists or pharmacists	48 (9.7)	31 (9.9)	0.94

Values are expressed as n (%). CKD: chronic kidney disease; eGFR: estimated glomerular filtration rate; GP: general practitioner; Q: question; SGLT2: sodium–glucose cotransporter 2. †p-values were calculated using the chi-square test. ^＊^Multiple responses were allowed where indicated. #Significant differences were observed after Bonferroni correction.

**Figure 2.**
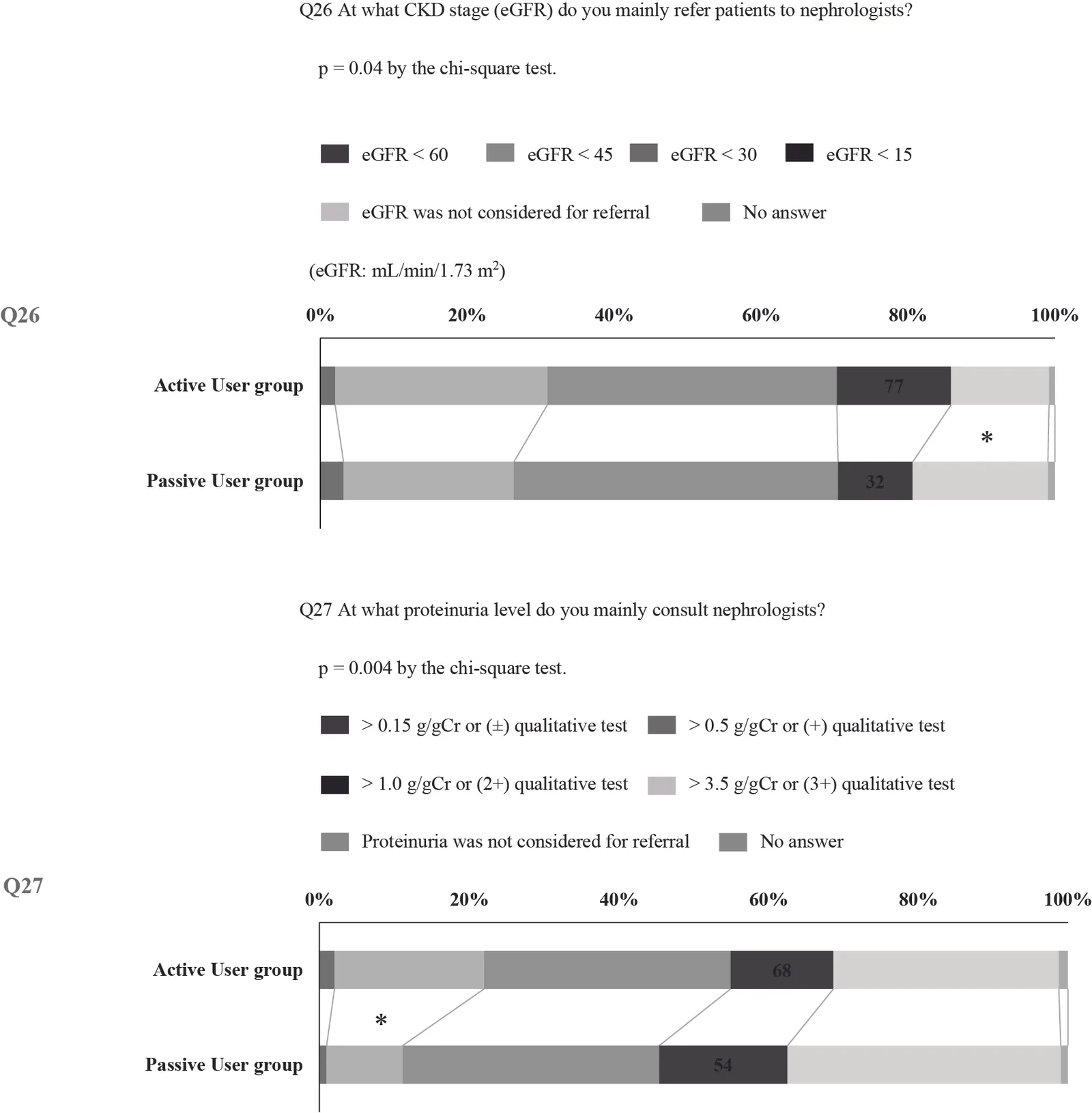
CKD level to consider for referral according to the utilization of SGLT2 inhibitors (Q26 and Q27). ^＊^Significant differences were observed after Bonferroni correction. Abbreviations: CKD, chronic kidney disease; eGFR, estimated glomerular filtration rate; SGLT2, sodium–glucose cotransporter 2.

### Results of logistic regression analysis

Table 4 presents the results of the logistic regression analysis identifying independent variables associated with the Active User group. Specialization in cardiology, allergy, use of serum cystatin C to evaluate kidney function, utilization of urine testing, use of eGFR and kidney imaging to define CKD, frequent assessment of quantitative albuminuria, use of hemoglobin levels to evaluate renal anemia, use of transferrin saturation levels to assess renal anemia, prescription of HIF-PH inhibitors, implementation of dietary therapy for patients with non-dialysis CKD, referral to nephrologists for both proteinuria and hematuria, and consultation for proteinuria >1.0 g/gCr or ≥2+ by qualitative testing were all positively associated with the Active User group. The corresponding odds ratios (95% confidence intervals [CIs]) were 1.84 (1.06-3.19), 6.13 (1.74-21.56), 2.05 (1.18-3.60), 34.8 (2.30-527.49), 1.96 (1.04-3.71), 4.52 (1.31-15.66), 1.72 (1.03-2.87), 2.10 (1.28-3.45), 2.56 (1.37-4.76), 2.03 (1.29-3.19), and 2.62 (1.19-5.75), respectively.

**Table 4.** Results of Logistic Regression Analysis Identifying Independent Factors Associated with the Active User Group.

Question		Answer	Reference group	p Value	Exp(B)	95 % CI
Specialty	Cardiology	Yes	No	0.03	1.84	[1.06-3.19]
	Rheumatology	Yes	No	0.042	0.28	[0.08-0.96]
	Allergy	Yes	No	0.005	6.13	[1.74-21.56]
Q2	Use of serum cystatin C level to evaluate kidney function	Yes	No	0.011	2.05	[1.18-3.60]
Q3	Diagnosis of CKD by both urine test and eGFR level	By both urine test and eGFR level	Only by urine test	0.042	15.55	[1.11-218.96]
	Diagnosis by urine test, eGFR level, and kidney imaging	By urine test, eGFR level, and kidney imaging examination	Only by urine test	0.01	34.80	[2.30-527.49]
Q5	Occasional quantitative testing for albuminuria	Occasional check quantitative tests for albuminuria	No quantitative testing	0.031	1.67	[1.05-2.67]
	Frequent quantitative testing for albuminuria	Frequent check of quantitative tests for albuminuria	No quantitative testing	0.039	1.96	[1.04-3.71]
Q9	Estimation of daily salt intake by 24-h urine collection	Yes	No	0.001	0.04	[0.006-0.23]
Q10	Use of hemoglobin level to evaluate renal anemia	Yes	No	0.017	4.52	[1.31-15.66]
	Use of transferrin saturation (TSAT) to evaluate renal anemia	Yes	No	0.038	1.72	[1.03-2.87]
Q12	Prescribes HIF-PH inhibitors at their own discretion	To prescribe HIF-PH inhibitors with my own discretion	Does not prescribe HIF-PH inhibitors	0.003	2.10	[1.28-3.45]
Q19	Provides dietary therapy for patients with CKD	To provide dietary therapy at my clinics/hospitals	Does not provide dietary therapy	0.003	2.56	[1.37-4.76]
Q25	Refers patients with evident proteinuria (> 0.5 g/gCr) to nephrologists	Yes	No	0.017	0.57	[0.36-0.90]
	Refers patients with both proteinuria and hematuria to nephrologists	Yes	No	0.002	2.03	[1.29-3.19]
Q27	Consults nephrologists for proteinuria > 1.0 g/gCr or (2+) by qualitative test	>1.0g/gCr or (2+) by qualitative proteinuria test	> 0.15 g/gCr or (±) by qualitative test	0.016	2.62	[1.19-5.75]

BP: blood pressure; CI: confidence interval; CKD: chronic kidney disease; eGFR: estimated glomerular filtration rate; HIF-PH: hypoxia-inducible factor prolyl hydroxylase; Q: question.

## Discussion

Our previous survey demonstrated that, while 83.3% of nephrologists reported prescribing SGLT2 inhibitors for CKD regardless of diabetes status, only 60.8% of non-specialists did so (p < 0.001) ^(12)^. Although SGLT2 inhibitors are now established as a core component of CKD therapy alongside RAS inhibitors, their adoption outside nephrology remains suboptimal. Further, this nationwide survey highlights a substantial implementation gap in CKD management, even among non-nephrologists.

Our post-hoc logistic regression analysis restricted to non-nephrologists revealed that SGLT2 inhibitor prescribing reflects an integrated approach to CKD management rather than an isolated therapeutic choice. The “active prescriber” profile was associated not with patient demographics but with physician specialty and practice patterns, particularly cardiology, rheumatology, and allergy, and with comprehensive diagnostic and management behaviors. These included the use of serum cystatin C, monitoring of systematic albuminuria in diabetes, application of explicit CKD diagnostic criteria, estimation of salt intake via 24-hour urine collection, assessment of anemia using hemoglobin and transferrin saturation levels, use of HIF-PH inhibitors, provision of dietary therapy, and adherence to structured referral criteria. Collectively, these behaviors demonstrate high “nephrology literacy”—the ability to identify, monitor, and manage CKD in an integrated and guideline-aligned manner. Although age distribution was not identified as an independent factor by the logistic regression analysis, a significant correlation was found between the age distribution and the two groups. Our results suggest that, in Japan, age and experience of physician may be important barriers to SGLT2 inhibitor adoption, rather than patient risk profile. This highlights the need for strategies tailored to physician demographics to improve appropriate utilization of these agents ^(14)^.

Our findings are consistent with global reports demonstrating persistently low adoption of SGLT2 inhibitors, despite robust evidence of their kidney and cardiovascular benefits. Baseline use in major trials remained modest, ranging from 4.1% among patients eligible for EMPA-KIDNEY trial to 14.4% in CREDENCE-eligible cohorts, with real-world data indicating treatment rates below 10% in many countries ^(14)^. In an analysis of 28 United States (U.S.) health systems using the Optum Labs Data Warehouse between 2022 and 2023, only 11.9% of patients with diabetes and a class 1A indication for SGLT2 inhibitor therapy (i.e., atherosclerotic cardiovascular disease, chronic kidney disease, or heart failure) received an SGLT2 inhibitor prescription. Among patients without diabetes but with a class 1A indication (urine albumin-to-creatinine ratio ≥200 mg/g or heart failure), the prescription rate was 3.1%, with subgroup rates of 1% in those with albuminuric CKD only and 4% in those with heart failure with or without albuminuric CKD ^(15)^. Similarly, Canadian real-world data indicated that fewer than 8% of patients with diabetes received SGLT2 inhibitors, and only 7% of those with concurrent CKD and diabetes were prescribed this evidence-based therapy ^(16)^. Previous literature has suggested that both patient-level factors (e.g., older age and frailty) and clinician-level factors (e.g., uncertainty regarding safety in complex patients and therapeutic inertia) contribute to the underuse of SGLT2 inhibitors in clinical practice ^(17)^. However, this survey was not designed to directly evaluate the effects of age or other demographic characteristics of physicians on prescribing behavior. Further research is warranted to elucidate how physician demographics, clinical setting, and the history of nephrology training, including duration, training level, and contents, influence the adoption of SGLT2 inhibitors in Japan.

The strong association between cardiology and active SGLT2 inhibitor prescribing reflects cardiologists’ increasing leadership in cardio–renal–metabolic medicine. Evidence from cardiovascular outcome and heart failure trials has reframed kidney protection as a central component of cardiovascular risk management ^(18)^. For cardiologists, SGLT2 inhibitors have become standard therapy for heart failure irrespective of diabetes status, and this familiarity with the drug class appears to extend naturally to renal protection. The novel association with rheumatology and allergy may suggest a shared conceptual framework centered on systemic inflammation and vigilant organ monitoring. Rheumatologists and allergists routinely evaluate kidney function in patients exposed to nephrotoxic agents or immune-mediated diseases ^(19)^, and their ongoing attention to subclinical organ injury likely facilitates earlier recognition of CKD and timely initiation of renoprotective therapy such as SGLT2 inhibitors.

Under-recognition of CKD and imprecise risk stratification remain major barriers to SGLT2 inhibitor use among non-nephrologists, and our data confirms that diagnostic precision drives therapeutic decision-making. Routine measurement of albuminuria in patients with diabetes was one of the strongest predictors of prescribing, underscoring its dual role as both a diagnostic and therapeutic indicator ^(20), (21)^. Physicians who do not assess albuminuria systematically risk overlooking early CKD and missing opportunities for timely SGLT2 inhibitor initiation. The use of serum cystatin C was another independent predictor. Cystatin C enables more accurate estimation of kidney function, particularly when reduced muscle mass distorts creatinine-based eGFR ^(22)^, and its use indicates clinical attentiveness to subtle renal impairment. Such diagnostic sophistication may represent a critical foundation for appropriate prescribing.

Active prescribers also exhibited a holistic approach that encompassed both lifestyle and hemodynamic management. Regular estimation of 24-hour urinary sodium excretion and implementation of dietary counseling indicates a strong commitment to individualized CKD management. Although seldom performed in routine clinical practice, 24-hour sodium measurement reflects advanced engagement with hemodynamic risk. This is clinically relevant because excessive dietary salt intake attenuates the hemodynamic benefits of SGLT2 inhibition, which depend partly on the restoration of tubulo-glomerular feedback and reduction of intra-glomerular pressure ^(23)^. Non-nephrologists who monitor salt intake appear to conceptualize SGLT2 inhibitors as components of an integrated therapeutic strategy rather than as isolated pharmacologic interventions.

Routine evaluation of hemoglobin and transferrin saturation, together with the use of HIF-PH inhibitors, was also associated with active prescribing. These behaviors characterize physicians who actively manage renal anemia and remain up to date with emerging CKD therapeutics ^(24)^. Non-nephrologists who are comfortable prescribing HIF-PH inhibitors demonstrate advanced familiarity with nephrology guidelines and CKD pathophysiology. This pattern suggests that early adopters of one novel therapy, such as HIF-PH inhibitors, are also more likely to adopt others, including SGLT2 inhibitors. In practice, the use of SGLT2 inhibitors therefore reflects broader competence in CKD management, rather than mere willingness to prescribe a new drug class.

Structured referral patterns also predicted active prescribing. Non-nephrologists who referred patients based on explicit thresholds for proteinuria or combined proteinuria and hematuria were significantly more likely to prescribe SGLT2 inhibitors. This finding indicates that systematic, criteria-based referral fosters proactive, rather than reactive, CKD management. Such non-nephrologists likely align their practice with domestic and international CKD guidelines, which recommend early nephrology referral for marked proteinuria or suspected glomerular disease ^(25), (26)^. Protocol-driven referrals may also enhance physicians’ confidence in initiating SGLT2 inhibitors, promoting timely collaborative management, and reducing therapeutic inertia.

Taken together, these findings suggest that active SGLT2 inhibitor prescribing by non-nephrologists reflects an integrated model of CKD care characterized by diagnostic rigor and multimodal management. This “active prescriber” profile is consistent with KDIGO 2024 guidance, which positions SGLT2 inhibitors within structured care pathways and ongoing patient monitoring ^(26)^. In this context, SGLT2 inhibitor adoption appears to be less an isolated prescribing decision than a downstream marker of overall CKD care quality. Experience from CKD care indicates that educational interventions, although essential, need to be embedded within broader multifaceted strategies to overcome the numerous patient-, provider-, and system-level barriers that impede implementation of guideline-directed therapies ^(27)^. Evidence from other health-system interventions further suggests that multifaceted quality-improvement programs, including academic detailing, clinical pharmacist support, and systematic identification of eligible patients, can achieve substantially greater gains in SGLT2 inhibitor uptake ^(28)^.

Future strategies to enhance SGLT2 inhibitor adoption in Japan should shift from product-focused promotion to a nephrology-literacy model that strengthens the foundations of primary care. Non-nephrologists should regard SGLT2 inhibitor initiation as a natural extension of structured CKD management, rather than as an isolated therapeutic intervention. Educational initiatives for non-specialists should therefore emphasize on comprehensive early CKD management, including a standardized “urine-first” strategy that uses albuminuria measurement to identify eligible patients who are often overlooked when clinicians rely solely on eGFR. Training should also include practical tools for estimating salt intake, such as spot urine–based formulas, to optimize the hemodynamic benefits of SGLT2 inhibitors, together with clear, streamlined protocols for sick-day rules and management of eGFR declines to reduce safety concerns and hesitation among primary care physicians.

In parallel, strengthening regional CKD care networks by leveraging Japan’s family doctor system ^(29)^ will be essential. Shared-care frameworks employing a Two-Attending Physician model could enable nephrologists to provide periodic oversight while primary care physicians continue maintenance therapy, including SGLT2 inhibitors, in routine clinical practice. Establishing secure e-consultation systems would allow non-nephrologists to obtain rapid guidance regarding initial eGFR declines or suspected adverse effects, thereby reducing therapeutic inertia without necessitating formal referrals. Furthermore, outpatient care should more fully integrate pharmacists and nurses into CKD management, drawing on lessons from models such as the U.S. Veterans Affairs system. Community pharmacists, in particular, could systematically identify patients already receiving RAS inhibitors but not yet treated with SGLT2 inhibitors despite clear eligibility, thereby prompting timely physician reassessment. By reframing the objective from “prescribing a drug” to “implementing a care system,” these coordinated strategies may help close the implementation gap and translate the proven benefits of SGLT2 inhibitors into improved kidney and cardiovascular outcomes across Japan.

### Study limitations

Several limitations of this study should be acknowledged. First, although the questionnaire was distributed to all 13,014 members of the Japan Physicians Association, the response rate was approximately 7.7%, introducing potential selection bias toward physicians with a greater interest in CKD or diabetes management. Nephrologists play a leading role in regional collaboration, and a larger number of specialists may be advantageous for regional collaboration, which may lead to proper and active prescribing of SGLT2 inhibitors for patients with CKD. Supplementary Figure 1 demonstrated the relationship between the ratio of Active User group among respondent GPs in Japan and the number of nephrologists per 100,000 people in each prefecture of Japan that was calculated using data of a number of nephrologists ^(30)^ and population by prefecture in Japan ^(31)^. No significant correlation was observed between these two factors. The low response rate and the bias among respondent GPs might lead to these results. Although our survey was nationwide, the number of respondent GPs was only about 1,000, and they might not be representative for all GPs in Japan. Also, due to the nature of the questionnaire survey, there is a tendency that GPs who are interested in CKD to respond to our questionnaires. Furthermore, this survey did not ask about the size of the medical area where the respondents work. More respondents and detailed questions are needed to clarify the relationship between active use of SGLT2 inhibitor for patients with CKD and the regional number of nephrologists.

Second, all data were self-reported and may not fully reflect actual prescribing behaviors or clinical practices in routine settings, creating the possibility of social desirability and recall bias. Furthermore, since we did not collect detailed information such as questions about the history of nephrology training, we cannot deny the possibility that unknown factors not collected in this survey influenced the results.

Third, because this was a cross-sectional survey, causal relationships between physician behaviors and active SGLT2 inhibitor prescribing cannot be inferred. It remains uncertain whether these practice patterns promote higher SGLT2 inhibitor use or whether early adopters subsequently refine their CKD management approaches.

Fourth, the analysis focused on members of a single national organization of GPs in Japan, which may limit the generalizability of findings to other types of providers (e.g., hospitalists, community specialists, or non-member physicians) and to healthcare systems outside Japan.

Fifth, management or treatment differ depending on the severity of CKD. Questionnaires that were used in this survey covered general content regarding CKD; however, they did not include questions specific to severity. Future surveys are needed to clarify how CKD management or treatment, including SGLT2 inhibitors, change among non-nephrologists depending on the severity of CKD.

Finally, although the logistic regression identified some significant practice-related predictors among non-nephrologists, unmeasured confounders, such as institutional policies, availability of nephrology consultation, or local educational initiatives, may also influence prescribing behavior.

## Conclusion

This nationwide survey shows that, among non-nephrologists in Japan, difference in the active prescription of SGLT2 inhibitors for patients with CKD was associated with a comprehensive and guideline-based approach to CKD management. The findings from this survey suggest that strategies to enhance SGLT2 inhibitor adoption should prioritize strengthening nephrology literacy and care pathways among non-nephrologists, rather than focusing solely on drug-centered education.

## Article Information

### Acknowledgments

We thank all members of the Japan Physicians Association who participated in this study. We are especially grateful to M. Miyazaki for organizing the survey and to K. Kobayashi for assistance with data collection and aggregation.

### Author Contributions

All authors read and approved the final manuscript. The main contributions of the authors are as follows: Satoru Tatematsu, Kazuo Kobayashi, Tsuguru Hatta, Taisuke Isozaki, Yosuke Nakayama, Junko Imura, Toshimasa Takahashi, Munehiro Kitada, and Yasunori Utsunomiya designed the study. Satoru Tatematsu, Kazuo Kobayashi, Tsuguru Hatta, Taisuke Isozaki, Yosuke Nakayama, Junko Imura, Toshimasa Takahashi, Munehiro Kitada, and Yasunori Utsunomiya collected the study data. Satoru Tatematsu, Kazuo Kobayashi, Tsuguru Hatta, Taisuke Isozaki, Yosuke Nakayama, Junko Imura, Toshimasa Takahashi, Munehiro Kitada, and Yasunori Utsunomiya performed the statistical analysis. Satoru Tatematsu, Kazuo Kobayashi, Tsuguru Hatta, Yosuke Nakayama, and Yasunori Utsunomiya were the major contributors to writing the manuscript.

### Conflicts of Interest

Kazuo Kobayashi has received lecture fees from Otsuka Pharmaceutical Co., Ltd. and Novartis Pharma. Tsuguru Hatta has received lecture fees from Otsuka Pharmaceutical Co., Ltd., Novartis Pharma, Eli Lilly Japan K.K., Boehringer Ingelheim, Daiichi Sankyo Co., Ltd., and Bayer Yakuhin, Ltd. Satoru Tatematsu, Taisuke Isozaki, Yosuke Nakayama, Junko Imura, Toshimasa Takahashi, Munehiro Kitada, and Yasunori Utsunomiya declare that there are no conflicts of interest.

### Compliance with Ethical Standards

#### Research Involving Human Participants and/or Animals

This study was conducted in accordance with the principles of the Declaration of Helsinki and was approved by the Ethical Review Board of the Japan Physicians Association on December 18, 2023 (Approval No. 0.3-2312-01).

### Informed Consent

The questionnaire was distributed among members of the Japan Physicians Association. Explanations regarding the purpose of the survey, the presentation of the results, and the preparation of the manuscript were clearly stated in the questionnaire. Submission of the completed questionnaire constituted informed consent for participation.

### Data Availability

The data supporting the findings of this study are stored in the repository of the Japan Physicians Association. The datasets generated and analyzed during this study are available from the corresponding author upon reasonable request.

## Supplement

Supplementary Material
