# Supplementary material for: Real-world Practice Patterns and Barriers to SGLT2 Inhibitor Use: A Nationwide Survey of Japanese General Practitioners
Source: JMA J. 2026 Aug 21;9(5):1296–309. doi: 10.31662/jmaj.2026-0197 (PMC13639645; doi:10.31662/jmaj.2026-0197)

Supplementary Data

Supplementary Table 1. Distribution of Responses Regarding the Utilization of SGLT2 Inhibitors in Patients with CKD

Question: Do you prescribe SGLT2 inhibitors to patients with conservative CKD?		Non-nephrologists (n = 835)	Nephrologists (n = 164)
Active User group	I prescribe them for patients with CKD regardless of whether or not proteinuria/albuminuria is present.	273 (32.7)	53 (31.5)
	I prescribe them to patients with CKD and positive proteinuria/albuminuria.	235 (28.1)	87 (51.8)
Passive User group	I prescribe them only to patients with diabetes and positive proteinuria/albuminuria.	144 (17.2)	9 (5.4)
	I do not prescribe them because patients are diagnosed as having CKD.	128 (15.3)	12 (7.1)
	I do not prescribe them.	44 (5.3)	5 (3.0)
	No answer	11 (1.3)	2 (1.2)

Values are expressed as n (%).

Among the total 1003 respondents, 11 non-nephrologists and 2 nephrologists did not answer this question.

Abbreviations: CKD: chronic kidney disease; SGLT2: sodium–glucose cotransporter 2.

Supplementary Table 2. Breakdown of Covariates that was Used in the Logistic Regression Analysis for "Passive User Group"

Question		Answer	Type of variable
Back ground	Age distribution	20s	
		30s	
		40s	ordinal variable
		50s	
		60s	
		70 years and older	
	Workplace	Clinic	Nominal variable (yes/no)
		Hospital	Nominal variable (yes/no)
	History of nephrology training		Nominal variable (yes/no)
	Specialty	Internal medicine	Nominal variable (yes/no)
		Cardiology	Nominal variable (yes/no)
		Diabetology and metabolism	Nominal variable (yes/no)
		Gastroenterology	Nominal variable (yes/no)
		Pulmonology	Nominal variable (yes/no)
		Neurology	Nominal variable (yes/no)
		Hematology	Nominal variable (yes/no)
		Rheumatology	Nominal variable (yes/no)
		Allergy	Nominal variable (yes/no)
		Psychiatry	Nominal variable (yes/no)
Q1	Do you possess the CKD guidelines 2023?	Yes, and I use them frequently.	
		Yes, but I have not used them.	
		No, but I would like to obtain the CKD Guidelines 2024 for GPs.	ordinal variable
		I do not possess them and do not wish to obtain the CKD Guidelines 2024 for GPs.	
Q2	What parameters do you use to assess kidney function?	Serum creatinine level.	Nominal variable (yes/no)
		$eGFR_{creatinine}$ level.	Nominal variable (yes/no)
		Serum cystatin C level.	Nominal variable (yes/no)
		$eGFR_{cystatinC}$ level.	Nominal variable (yes/no)
		Creatinine clearance (24-h urine collection).	Nominal variable (yes/no)
Q3	How do you diagnose patients as having CKD?	By urine test, eGFR level, and kidney imaging.	
		By both urine test and eGFR level.	ordinal variable
		Only by eGFR level.	
		Only by urine test.	
Q4	Do you check urine tests for patients with CKD during regular visits?	Perform quantitative tests for proteinuria (g/gCr).	
		Perform only qualitative tests for proteinuria.	ordinal variable
		Do not perform urine testing.	
Q5	Do you check urine tests for patients with CKD during regular visits?	Quantitative tests every 3 months.	
		Quantitative tests once or twice a year.	ordinal variable
		Rarely perform quantitative tests.	
		Perform only qualitative tests.	
		Do not check albuminuria.	
Q6	Do you evaluate BP in patients with CKD during clinic visits?	I evaluate both office BP and home BP in the early morning.	
		I evaluate only home BP in the early morning.	ordinal variable
		I evaluate only office BP.	
		I do not evaluate either.	
Q7	Do you prescribe RAS inhibitors to patients with CKD and hypertension?	I prescribe them as first-line medication.	
		I prescribe them to patients with proteinuria.	ordinal variable
		I rarely prescribe them.	
Q8	When do you discontinue RAS inhibitors in patients with CKD?	When kidney function deteriorates.	Nominal variable (yes/no)
		When the serum Cr level reaches 2 mg/dL.	Nominal variable (yes/no)
		When the serum potassium level exceeds the normal range.	Nominal variable (yes/no)
		I continue prescribing them for their renoprotective effects.	Nominal variable (yes/no)
		I do not prescribe them to CKD patients.	Nominal variable (yes/no)
Q9	How do you evaluate salt intake in patients with CKD?	By Tanaka's formula using spot urine samples.	Nominal variable (yes/no)
		By direct measurement using 24-h urine collection.	Nominal variable (yes/no)
		By patient interview.	Nominal variable (yes/no)
		I do not evaluate salt intake by either method.	Nominal variable (yes/no)
Q10	Which parameters do you use to evaluate renal anemia?	Hemoglobin level.	Nominal variable (yes/no)
		Mean corpuscular volume (MCV).	Nominal variable (yes/no)
		Serum iron (Fe) level.	Nominal variable (yes/no)
		Transferrin saturation (TSAT).	Nominal variable (yes/no)
		Serum ferritin level.	Nominal variable (yes/no)
		Serum erythropoietin level.	Nominal variable (yes/no)
Q11	Do you prescribe erythropoiesis-stimulating agents to patients with CKD?	Yes, I prescribe them at my own discretion.	ordinal variable

		Yes, I prescribe them when recommended by a nephrologist.	
		No, I do not prescribe them.	
Q12	Do you prescribe HIF-PH inhibitors to patients with CKD?	Yes, I prescribe them at my own discretion.	
		Yes, I prescribe them when recommended by a nephrologist.	ordinal variable
		No, I do not prescribe them.	
Q13	What is your target hemoglobin level for patients with non-dialysis-dependent CKD?	> 12 g/dL	
		> 11 g/dL	ordinal variable
		Depending on each individual case	
		> 10 g/dL	
		> 9 g/dL	
Q14	What is your target ferritin level for patients with non –dialysis-dependent CKD?	> 25 ng/mL	
		> 100 ng/mL	ordinal variable
		Depending on each individual case	
		> 200 ng/mL	
		> 300 ng/mL	
Q16	How do you manage serum potassium levels in patients with CKD?	I regularly check potassium levels and manage hyperkalemia continuously.	
		I check potassium regularly but provide only temporary treatment.	
		I check potassium regularly but refer hyperkalemia management to a nephrologist.	ordinal variable
		I do not check potassium but manage hyperkalemia per nephrologist instructions.	
		I neither check nor treat hyperkalemia.	
Q17	At what serum potassium level do you initiate hyperkalemia treatment in non-dialysis-dependent CKD?	> 4.5 mEq/L	
		> 5.0 mEq/L	ordinal variable
		Depending on each individual case	
		> 5.5 mEq/L	
		> 6.0 mEq/L	
Q18	Do you evaluate serum calcium and phosphorus levels in patients with CKD?	I regularly check both calcium and phosphorus levels.	
		I regularly check only calcium levels.	ordinal variable
		I check them only in patients on vitamin D therapy.	
		I do not check them but rely on nephrologists.	
		I never check them.	
Q19	Do you provide dietary therapy to patients with non-dialysis-dependent CKD?	I provide it at my clinic or hospital.	
		I refer patients to nephrologists for dietary therapy.	ordinal variable
		I neither provide nor refer for dietary therapy.	
Q20	What types of CKD dietary therapy do you provide?	Restriction of daily salt intake.	Nominal variable (yes/no)
		Restriction of daily protein intake.	Nominal variable (yes/no)
		Guidance on appropriate daily calorie intake.	Nominal variable (yes/no)
		Restriction of daily phosphate intake.	Nominal variable (yes/no)
Q21	Is CKD collaborative care between GPs and nephrologists active in your region?	Yes, fully active	
		Yes, partially active	ordinal variable
		No, it does not work well	
		No, and there is no CKD collaborative care between GPs and nephrologists.	
Q22	Do you know any nephrologists whom you can consult regarding CKD patients?	Yes, I know several nephrologists available for consultation	
		Yes, I know only one nephrologist available for consultation	ordinal variable
		No	
Q23	How is your relationship with nephrologists available for consultation?	I know them well personally	
		I know only their names	ordinal variable
		I do not know them well	
Q24	Do you use the referral criteria for CKD consultation recommended in the CKD Guidelines (Japanese Society of Nephrology)?	I use it often	
		I use it sometimes	ordinal variable
		I rarely use them	
		I know about them but do not use them	
		I do not know about them	
Q25	For which CKD patients do you usually consult nephrologists?	With evident proteinuria (>0.5 g/gCr)	Nominal variable (yes/no)
		With both proteinuria and hematuria	Nominal variable (yes/no)
		With eGFR <45 mL/min/1.73 m^2	Nominal variable (yes/no)
		With rapid eGFR decline	Nominal variable (yes/no)
		With worsening symptoms including edema	Nominal variable (yes/no)
		With worsening control of blood pressure or glucose	Nominal variable (yes/no)
		With need for dietary counseling	Nominal variable (yes/no)
		With need for explanation of kidney replacement therapy or conservative kidney management	Nominal variable (yes/no)

Q26	At what CKD stage (eGFR) do you mainly refer patients to nephrologists?	eGFR < 60 mL/min/1.73 m^2	ordinal variable
		eGFR < 45 mL/min/1.73 m^2	
		eGFR not considered for referral	
		eGFR < 30 mL/min/1.73 m^2	
		eGFR < 15 mL/min/1.73 m^2	
Q27	At what proteinuria level do you mainly consult nephrologists?	> 0.15 g/gCr or ($\pm$) qualitative test	ordinal variable
		> 0.5 g/gCr or (+) qualitative test	
		Proteinuria not considered for referral	
		> 1.0 g/gCr or (2+) qualitative test	
		> 3.5 g/gCr or (3+) qualitative test	
Q28	What CKD-related treatments would you like nephrologists to provide?	Salt reduction counseling	Nominal variable (yes/no)
		CKD-specific dietary counseling	Nominal variable (yes/no)
		Management of blood pressure	Nominal variable (yes/no)
		Management of plasma glucose	Nominal variable (yes/no)
		Treatment for renal anemia	Nominal variable (yes/no)
		Treatment of electrolyte abnormalities or metabolic acidosis	Nominal variable (yes/no)
		Explanation of kidney replacement therapy or conservative kidney management	Nominal variable (yes/no)
Q29	Are you satisfied with nephrologists' CKD consultation?	Completely satisfied	ordinal variable
		Satisfied sometimes	
		Neither satisfied nor dissatisfied	
		Occasionally dissatisfied	
		Not satisfied at all	
Q30	Which aspects of the consultation were unsatisfactory?	Insufficient patient counseling or explanation	Nominal variable (yes/no)
		Insufficient feedback to GPs	Nominal variable (yes/no)
		Inadequate treatment (e.g., no change in medication)	Nominal variable (yes/no)
		Insufficient counseling by nutritionists or pharmacists	Nominal variable (yes/no)

Abbreviations: BP: blood pressure; CKD: chronic kidney disease; Cr: creatinine; eGFR: estimated glomerular filtration rate; GP: general practitioner; HIF-PH: hypoxia-inducible factor prolyl hydroxylase; Q: question; RAS: renin–angiotensin system; SGLT2: sodium–glucose cotransporter 2.

Supplementary Figure 1　Ratio of Active User Group by prefecture in Japan

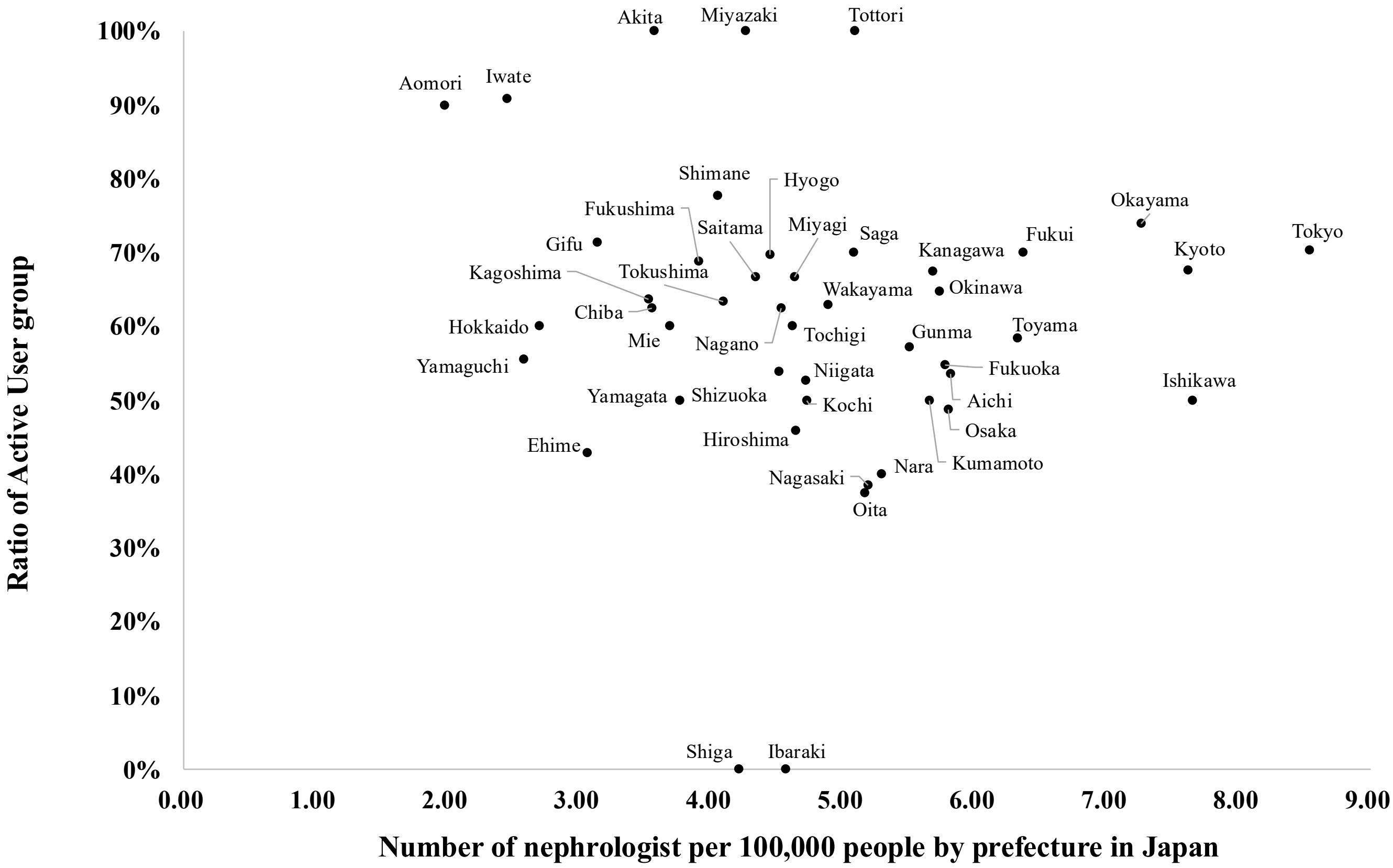

Supplement: Supplementary file 1 [file 2433-3298-9-5-1296-s001.pdf]
